# Pathogen detection by targeted next-generation sequencing test in adult hematological malignancies patients with suspected infections

**DOI:** 10.3389/fmed.2024.1443596

**Published:** 2024-09-24

**Authors:** Jin-Hui Xu, Ya-Bin Cui, Li-Jie Wang, Hui-Jie Nan, Pei-Yao Yang, Yan-Liang Bai, Ming-Yue Shi

**Affiliations:** ^1^Department of Hematology, Zhengzhou University People’s Hospital and Henan Provincial People’s Hospital, Zhengzhou, China; ^2^Department of Hematology, Henan University People’s Hospital and Henan Provincial People’s Hospital, Zhengzhou, China

**Keywords:** hematological malignancies, infections, targeted next-generation sequencing, conventional microbiological tests, pathogenic detection

## Abstract

**Background:**

Infections in patients with hematological malignancies (HM) are a significant cause of morbidity and mortality. Timely and effective empirical anti-infective treatment can reduce the infection-related mortality rate. Targeted next-generation sequencing (tNGS) offers a rapid diagnostic approach for identifying diverse pathogens in these patients. However, relevant research is still limited to adult patients with HM.

**Methods:**

We conducted a retrospective analysis of adult HM patients admitted to our hospital from March 2023 to September 2023, focusing on their clinical characteristics and the results of both tNGS and conventional microbiological tests (CMTs). We evaluated the performance of tNGS and CMTs in pathogenic diagnosis and described the distribution characteristics of pathogens in adult HM patients with infections.

**Results:**

The study included 209 samples collected from 137 patients. Results showed that the overall pathogen detection rate differed significantly between tNGS and CMTs (60.3% vs. 24.4%, *p* < 0.001). The sensitivity (69.7% vs. 35.9%), negative predictive value (NPV) (48.2% vs. 42.4%), and accuracy (66.5% vs. 56.5%) of pathogen detection were notably superior with tNGS compared to CMTs. Among the 142 samples with clinically diagnosed infections, tNGS combined with CMTs identified a definite or probable microbial etiology in 114 samples (80.3%). Of the 36 samples with concordant positive results from both tNGS and CMTs, 72.2% (26/36) exhibited full or partial agreement. Our study further showed the highest detection rate for viral infections (57.0%), predominantly for Epstein–Barr virus (DNA-V, 18.3%). Followed by bacterial infections (46.5%), the detection rate of Gram-negative bacteria (G^+^, 35.9%) was higher than that of Gram-positive bacteria (G^−^, 21.8%) in this study. *Klebsiella pneumoniae* (G^−^, 12.7%) had the highest detection rate among these emerging bacteria, followed by *Pseudomonas aeruginosa* (G^−^, 10.6%) and *Enterococcus faecium* (G^+^, 7.7%). Bacterial-viral coinfections were the most common type of mixed infection (35.5%).

**Conclusion:**

In conclusion, tNGS outperforms CMTs in both sensitivity and pathogen spectrum. Therefore, it can serve as an adjunct to CMTs to facilitate the precise adjustment of anti-infective regimens for adult HM patients. Our findings establish a basis for formulating empirical anti-infective therapy strategies tailored to the pathogen distribution in this patient population.

## 1 Introduction

Hematological malignancies (HM) originate from the hematopoietic system and are characterized by abnormalities such as anemia, bleeding, and susceptibility to infections (1, 2). These malignancies mainly comprise leukemia, lymphoma, multiple myeloma, and myelodysplastic syndromes. In over 80% of patients with hematological cancers, infection commonly arises as a complication due to the primary disease, intensive radiotherapy, chemotherapy, immunotherapy, and transplantation (2–4). Regarded as a medical emergency, it necessitates immediate evaluation and the administration of empiric broad-spectrum antibiotics (5). Often, initial clinical symptoms in these patients are subtle, with most showing only fever. Both the pathogens and the sites of infection remain unclear (5). Furthermore, HM has a notably high infection-related mortality rate, predominantly due to bloodstream infections (42%) (6). Consequently, administering effective empiric anti-infective treatments is crucial to lower the infection-related mortality rate (7). Nevertheless, the ineffectiveness of empirical treatments still results in some patients succumbing to infections. Unnecessary antibiotic use not only fosters drug resistance but also reduces therapeutic effectiveness (7). Clinicians currently lack effective methods for timely adjustment of antibiotic treatments.

CMTs encompass a variety of diagnostic techniques including conventional microscopy, culturing, biochemical testing, polymerase chain reaction (PCR), and immunology technology. While conventional microscopy, culturing, and biochemical testing remain the gold standards for the detection of bacterial, fungal, and parasitic pathogens (8), culturing may have limitations in sensitivity for slow-growing, fastidious, or uncultivable microorganisms, especially in the presence of prior antimicrobial therapy (9). Molecular diagnostic methods, such as immunoassays and PCR assays, offer alternative options but are often limited to detecting a predetermined range of pathogens (10, 11). To improve the diagnostic process for infections in adult patients with HM, additional tools are required (12). Emerging as a technology with notable potential, tNGS addresses the limitations of metagenomic next-generation sequencing (mNGS) by identifying a broad spectrum of pathogens. Earlier investigations have underscored that microbial next-generation sequencing provides comprehensive and relatively unbiased pathogen information quickly and with high throughput for patients with hematologic disorders (13). However, the broad clinical application of mNGS is limited by its high cost, susceptibility to interference from host nucleic acids, and the need for separate detection of DNA and RNA detection. tNGS, combined with high-throughput sequencing and ultra-multiplex PCR, allows for the simultaneous detection of multiple common pathogens. While tNGS detects fewer pathogens than mNGS, it provides significant advantages in cost and diagnostic efficiency (14). A preliminary report indicates that tNGS detects pathogens at just one quarter of mNGS’s cost, demonstrating its effectiveness (15). Additionally, another study has shown that tNGS matches mNGS in diagnostic performance for microbiological testing (16, 17).

This study endeavors to elucidate the potential utility of tNGS in the pathogenic diagnosis of HM patients with suspected infections. We utilized a tNGS assay targeting 207 pathogens (Supplementary Table S1) to evaluate its clinical performance in comparison with CMTs. Furthermore, we aimed to elucidate the distribution of pathogens within the study population based on the results from tNGS and CMTs.

## 2 Materials and methods

### 2.1 Study samples

We retrospectively reviewed 209 samples (137 patients) with HM and suspected infection at Henan Province People’s Hospital between March 2023 and September 2023. With our inclusion criteria, 209 samples were enrolled for this study. The inclusion criteria were as follows, (i) diagnosed with hematological malignancy, (ii) single axillary temperature ≥38°C, or axillary temperature ≥37.7°C for more than 1 h, (iii) completed the CMTs, such as smears, cultures, and PCR tests, and the CMTs and tNGS were tested no more than 48 h apart, (iv) complete clinical history. Additionally, we gathered clinical characteristics from the samples, including clinical symptoms, laboratory test results, imaging examination results, diagnosis, treatment process, and prognosis.

### 2.2 Targeted next-generation sequencing

#### 2.2.1 Preprocessing of clinical samples

To liquefy the sputum sample, add an equal volume of 1% dithiothreitol (DTT) and mix thoroughly by shaking until the sample becomes non-viscous. From this mixture, 600 μL is taken for nucleic acid extraction. Transfer 2.0 mL of the bronchoalveolar lavage fluid and pleural effusion treatment into a 2.0 mL centrifuge tube and centrifuge at 13,000 rpm for 10 min. Discard the supernatant and retain 600 μL for nucleic acid extraction. Centrifuge the peripheral blood sample at 13,000 rpm for 10 min to separate the plasma from the cellular components. Carefully remove the upper layer of plasma and retain the cell pellet, taking 600 μL for nucleic acid extraction. To the extracted sample, add 2 g of 0.5 μm glass beads along with an appropriate amount of lytic enzyme to facilitate complete lysis of microorganisms and the release of nucleic acids.

#### 2.2.2 Nucleic acid (DNA/RNA) extraction

Nucleic acids (DNA/RNA) are extracted using a nucleic acid co-extraction kit (DP307, Tiangen, Beijing, China), following the manufacturer’s protocol. During the nucleic acid extraction process, a nucleic acid precipitation agent should be added to enhance extraction efficiency. The purity of the extracted nucleic acids is assessed using a Nanodrop spectrophotometer (Life Technologies). Typically, a ratio of A260/A280 greater than 1.8 and A260/A230 greater than 2.0 is considered indicative of high-purity nucleic acids.

#### 2.2.3 Design of multiplex pathogen targeted amplification primers

A total of 207 common pathogenic microorganisms (Supplementary Table S1) and 21 antibiotic-resistance genes were selected. Using the reference sequences of representative genomes of these pathogens, specific non-conserved sequences were identified through BLAST software. Based on these non-conserved sequences, primers specific for the identification of pathogenic microorganisms were designed. The designed primers were synthesized by a commercial provider.

#### 2.2.4 Library construction and sequencing

Initially, the optimized primer combinations were used to enrich and amplify the pathogenic microorganisms present in clinical samples. The resulting amplicons were purified using magnetic beads to remove primer dimers and impurities. The purified amplicons underwent a second round of PCR amplification, during which sequencing adapters (including sequencing primers and indexes) were added. After another round of magnetic bead purification, the sequencing library was obtained. The normal size of library fragments is approximately 350 bp, as assessed by the Agilent 2100 Bioanalyzer (Agilent Technologies, Santa Clara, CA, United States). There should be no significant primer dimers or non-specific amplification bands, and a library concentration greater than 1 ng/μL is considered acceptable. The constructed multiplex pathogen libraries were mixed in equal quantities. DNBs (DNA nanoballs) were prepared using the DNBSEQ One-step DNB Preparation Kit (1000026466, Wuhan MGI Tech Co., Wuhan, China) and sequenced on the MGISEQ-200 platform using the single-end 75 bp sequencing mode. For each sample, the FastQ data output should not be fewer than 0.1 million reads.

#### 2.2.5 Data analysis

After sequencing, the obtained amplicons were processed to remove low-quality reads and those with a length of less than 60 bp. The remaining high-quality amplicons were aligned against the human genome to eliminate any contamination from human-derived sequences. This process resulted in the acquisition of high-quality amplicons specific to pathogenic microorganisms. Subsequently, these high-quality amplicons were mapped back to the designed target regions to identify the species of the pathogenic microorganisms present in the samples.

### 2.3 Conventional microbiological tests

The CMTs included bacterial and fungal smears and cultures, as well as real-time PCR conducted with real-time PCR systems for the amplification and quantification of viral nucleic acids, such as Epstein–Barr virus, cytomegalovirus, polyomavirus BK, polyomavirus JC, and severe acute respiratory syndrome coronavirus 2. Additionally, serum antibody tests were performed using indirect immunofluorescence assays to detect pathogens including *Legionella pneumophila*, *Mycoplasma pneumoniae*, *Coxiella burnetii*, *Chlamydophila pneumoniae*, adenovirus, respiratory syncytial virus, influenza A, influenza B and parainfluenza 1, 2 and 3 (Table 1).

**Table 1:** Baseline characteristics of the 209 samples enrolled.

Sample characteristic	Value (*n* = 209)
Median age [median (IQRs)]	59 (49.0–66.0)
Gender, *n* (%)	
Male	105 (50.2)
Female	104 (49.8)
Febrile neutropenia, *n* (%)	
Yes	92 (44.0)
No	117 (56.0)
Basic disease, *n* (%)	
Leukemia	124 (59.3)
Lymphoma	50 (23.9)
Myelodysplastic syndromes	16 (7.7)
Multiple myeloma	19 (9.1)
Sample, *n* (%)	
Peripheral blood	140 (67.0)
Sputum	61 (29.2)
BALF	4 (2.0)
Parapneumonic effusions	2 (1.0)
Cerebrospinal fluid	1 (0.5)
Urine	1 (0.5)
Previous treatment, *n* (%)	
Transplantation	22 (10.5)
Non-transplantation	187 (89.6)
Antibiotic exposure, *n* (%)	
Yes	193 (92.3)
No	16 (7.7)
Change antimicrobial regimen, *n* (%)	
Yes	88 (42.1)
No	121 (57.9)
Mean total of cost [median (IQRs)]	71,092 (34081.5–99337.0)
Length of stay [median (IQRs)]	25 (14–32.5)

BALF, broncho-alveolar lavage fluid.

### 2.4 Interpretation of tNGS results

Consistent with the experimental approach of targeted microbial sequence amplification using specific primers, the normalized read count of detected microorganisms in the sample served as the primary interpretive indicator. To classify a microorganism as a potential pathogen, specific criteria were defined (14).

- bacteria (excluding *Mycobacterium tuberculosis complex*), fungi and atypical pathogen: normalized read count ≥10;
- viruses: normalized read count ≥10;
- *Mycobacterium tuberculosis complex*: normalized read count ≥1.

### 2.5 Clinical evaluation

All tNGS tests were conducted upon request by hematology specialists, and patients were subsequently monitored by specialized hematology physicians specializing in HM cases. These physicians performed a thorough analysis of clinical data to assess the presence of infections and the clinical relevance of potential pathogens. The comprehensive evaluation included medical history, symptoms, imaging results, tNGS findings, and CMTs outcomes. Any discrepancies were resolved through consultation with a senior physician to reach consensus. The sensitivity and specificity of tNGS tests were determined based on their clinical relevance. Additionally, we assessed tNGS diagnostic performance in comparison to clinical diagnosis, including positive predictive value (PPV), negative predictive value (NPV), specificity, and sensitivity. We also assessed the agreement between tNGS and CMTs. “Complete consistency” was noted if both methods detected identical pathogens, while “Complete inconsistency” referred to completely different findings. “Partial consistency” indicated partial agreement between the detected pathogens. Mixed infection was defined as the presence of multiple pathogens in a single infection. This study aims to describe the distribution of pathogens in samples from adult HM patients with infections. Consequently, at least two seasoned hematology physicians treating each patient made decisions regarding the clinical significance of the identified pathogens. There was no retrospective reassessment or reevaluation by a third party or committee.

### 2.6 Statistical analysis

Statistical analysis was performed using SPSS version 25.0 software. Agreement measures, including percent positive agreement (PPA), percent negative agreement (PNA), and percent overall agreement (POA), between tNGS and CMTs were calculated. Sensitivity, specificity, PPV, NPV, and accuracy for tNGS and CMTs tests were determined following a clinical data review, which classified infections as proven, probable, possible, or unlikely. Normally distributed data were presented as mean ± standard deviation and analyzed using the *t*-test. Non-normally distributed data were reported as median (interquartile range) [M (P25, P75)] and analyzed with the Mann–Whitney *U* test. Comparative analysis was performed using Pearson’s *χ*^2^ test. *p*-values of <0.05 were considered significant.

## 3 Result

### 3.1 Characteristics of the samples from adult HM patients with suspected infections

In this study, we reviewed and potentially enrolled 286 samples from 162 patients diagnosed with HM and undergoing tNGS. Seventy-seven samples were excluded for various reasons, including the lack of paired CMTs (*n* = 68), incomplete data (*n* = 2), and the presence of aplastic anemia (*n* = 7). As a result, 209 samples from 137 patients who met the enrollment criteria formed the definitive cohort and underwent further analysis. The reason for having more samples than patients is due to multiple samples being collected from some patients at different time points to detect infectious pathogens over various periods. The median age of the cohort was 59 years. There was no difference in the proportion of female and male samples (49.8% vs. 50.2%). A total of 92 (44.0%) samples exhibited febrile neutropenia (FN). The samples exhibited a wide variety of underlying diseases and sample types. The most common underlying diseases were leukemia (59.3%), followed by lymphoma (23.9%), multiple myeloma (7.7%), and myelodysplastic syndromes (9.1%). The samples included peripheral blood, sputum, bronchoalveolar lavage fluid (BALF), parapneumonic effusions, cerebrospinal fluid, and urine. Peripheral blood (67.0%) and sputum (29.2%) samples were the most common types of tNGS. Twenty-two (10.5%) samples had undergone hematopoietic stem cell transplantation (HSCT). A majority of samples (193, 92.3%) had been exposed to antibiotics before sample collection. A total of 75 (35.9%) samples changed the antibiotic regimen after the detection of tNGS and CMTs. The mean total cost was 71,092 yuan, and the median length of stay was 25 days. Adult HM patients with suspected infections are at risk of significantly prolonged hospital stays and increased hospitalization costs (Table 1).

In the analysis of samples from 22 transplantation patients, 12 samples were collected during the transplantation period due to suspected infections. The remaining 10 samples were collected during readmissions more than 3 months post-transplantation, with five obtained within the first year and the other five collected over a year after transplantation. Among these 22 patients, three experienced a relapse of hematological malignancy following transplantation (Supplementary Table S2).

### 3.2 Comparison of results by tNGS and CMTs regardless of clinical relevance

A total of 61 potential pathogens were detected through tNGS, including 10 Gram-positive bacteria, 15 Gram-negative bacteria, 11 fungi, 9 DNA viruses, 12 RNA viruses, and 4 atypical pathogens. In comparison, CMTs identified 21 pathogens, including 4 Gram-positive bacteria bacteria, 10 Gram-negative bacteria, 1 fungus, 3 DNA viruses, 2 RNA viruses, and 1 atypical pathogen. There was no statistically significant difference in the distribution of bacterial, fungal, and viral pathogens between the two groups (*p* = 0.295) (Table 2; Supplementary Table S3).

**Table 2:** Detection result of tNGS and CMTs regardless of clinical relevance.

	tNGS, *n* (%)	CMTs, *n* (%)	*p*
Pathogens species	61	21	
Bacteria	25 (41.0)	14 (66.7)	
G^+^	10 (16.4)	4 (19.0)	
G^−^	15 (24.6)	10 (47.6)	
Fungi	11 (18.0)	1 (4.8)	0.295
Viruses	21 (34.4)	5 (23.8)	
DNA-V	9 (14.8)	3 (14.3)	
RNA-V	12 (19.7)	2 (9.5)	
AP	4 (6.6)	1 (4.8)	
Positive rate	126 (60.3)	51 (24.4)	<0.001^*^
Detection rate of the bacteria	64 (30.6)	23 (11.0)	<0.001^*^
G^+^	33 (15.8)	5 (2.4)	<0.001^*^
G^−^	46 (22.0)	19 (9.1)	<0.001^*^
Detection rate of the fungi	28 (13.4)	1 (0.5)	<0.001^*^
Detection rate of the viruses	93 (44.5)	30 (14.4)	<0.001^*^
DNA-V	67 (32.1)	16 (7.7)	<0.001^*^
RNA-V	41 (19.6)	14 (6.7)	<0.001^*^
Detection rate of the AP	5 (2.4)	3 (1.4)	0.473

^*^p-value <0.05. tNGS, targeted next generation sequencing; CMTs, conventional microbiological tests. G^+^, Gram-positive bacteria; G^−^, Gram-negative bacteria; DNA-V, DNA virus; RNA-V, RNA virus; AP, atypical pathogens.

Among the 209 enrolled samples, the detection rates of bacteria, fungi, and viruses were all significantly higher with tNGS compared to traditional methods (*p* < 0.001) (Table 2). Additionally, PPA, PNA, and POA between tNGS and CMTs were 70.6, 43.0, and 49.8%, respectively (Table 3). According to our data, the detection efficiency of tNGS was significantly higher than that of CMTs, and the positive rate appeared to be less affected by prior antibiotic usage.

**Table 3:** Comparison of the agreement of tNGS versus CMTs regardless of clinical relevance.

Test system	CMTs positive/statistics	CMTs negative/value
tNGS positive	36	90
tNGS negative	15	68
Total	51	158
	Percent positive agreement	70.6%
	Percent negative agreement	43.0%
	percent overall agreement	49.8%

tNGS, targeted next generation sequencing; CMTs, conventional microbiological tests.

**Figure 1:**
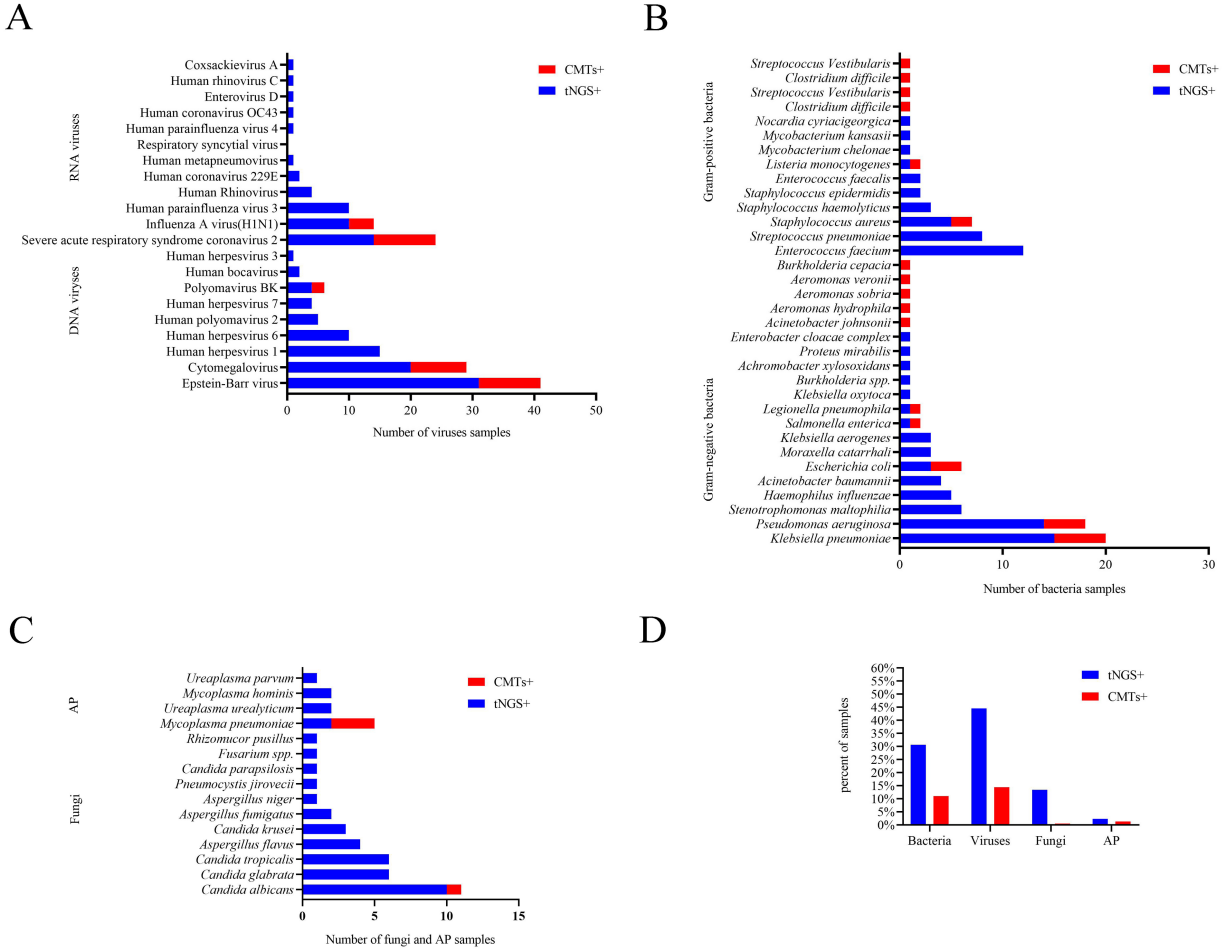
**(A–D)** Comparison of pathogens results by tNGS and CMTs. AP, atypical pathogens.

Among all samples, bacteria, fungi, viruses, and atypical pathogens were detected in 64/209 (30.6%), 28/209 (13.4%), 93/209 (44.5%), and 5/209 (2.4%) samples by tNGS, respectively (Table 2), and the most common species of the detected bacteria, fungi, and viruses were *Klebsiella pneumoniae*, *Candida albicans*, and Epstein–Barr virus, respectively (Figure 1). However, CMTs detected bacteria, fungi, viruses, and atypical pathogens in 23/209 (11.0%), 1/209 (0.5%), 30/209 (14.4%), and 3/209 (1.4%) samples, respectively (Table 2). The most common species of detected bacteria and viruses were *Klebsiella pneumoniae* and Epstein–Barr virus, which were similar to those identified by tNGS. *Candida* and *Mycoplasma pneumoniae* were the only fungi and atypical pathogens isolated by the conventional method (Figure 1).

In conclusion, seven microorganisms were solely identified via CMTs: *Burkholderia cepacia*, *Clostridium difficile*, *Streptococcus vestibularis*, *Aeromonas hydrophila*, *Aeromonas sobria*, *Aeromonas veronii*, and *Acinetobacter johnsonii*. Each of these microorganisms was detected in only one patient among the study population, and the latter six were beyond the detection capability of tNGS (Figure 1).

### 3.3 Comparison of clinical diagnostic performance between tNGS and CMTs

The comparison of the diagnostic performance between the tNGS test and CMTs is shown in Table 4. tNGS showed approximately 2-fold higher sensitivity (69.7% vs. 35.9%), higher NPV (48.2% vs. 42.4%), and the accuracy (agreement) of tNGS (66.5%, kappa = 0.277) was higher than CMTs (56.5%, kappa = 0.264). Furthermore, the detection results of tNGS and CMTs were consistent with the clinical diagnosis of the infection (*p* < 0.001). However, as expected, tNGS had lower specificity (59.7%), and lower PPV (78.6%) compared to CMTs. Furthermore, in the detection of bacteria and fungi, tNGS demonstrated sensitivities of 74.7 and 32.0%, respectively, with concordance rates of 82.3% (kappa = 0.614, *p* < 0.001) and 75.6% (kappa = 0.376, *p* < 0.001) when compared to microbial cultures. For viral detection, tNGS exhibited sensitivities of 88.5 and 38.5% in comparison to CMTs (virus), with concordance rates of 84.2% (kappa = 0.675, *p* < 0.001) and 77.0% (kappa = 0.439, *p* < 0.001). Overall, the detection results of tNGS for bacteria, fungi, and viruses demonstrated a high level of agreement with the clinical diagnoses of infections (Supplementary Tables S4, S5). This indicates that tNGS is a more reliable method for describing the distribution of pathogen infections.

**Table 4:** Diagnostic test evaluation of 209 samples with tNGS results.

Test system	Infection present/statistics	Infection not present/value
tNGS positive	99	27
tNGS negative	43	40
CMTs positive	51	0
CMTs negative	91	67
tNGS		
	Sensitivity	69.7%
	Specificity	59.7%
	Disease prevalence	67.9%
	Positive predictive value	78.6%
	Negative predictive value	48.2%
	Accuracy (agreement)	66.5%
	Kappa	0.277^*^
CMTs		
	Sensitivity	35.9%
	Specificity	100.0%
	Disease prevalence	67.9%
	Positive predictive value	100.0%
	Negative predictive value	42.4%
	Accuracy (agreement)	56.5%
	Kappa	0.264^*^

^*^p-value <0.001. tNGS, targeted next generation sequencing; CMTs, conventional microbiological tests.

**Figure 2:**
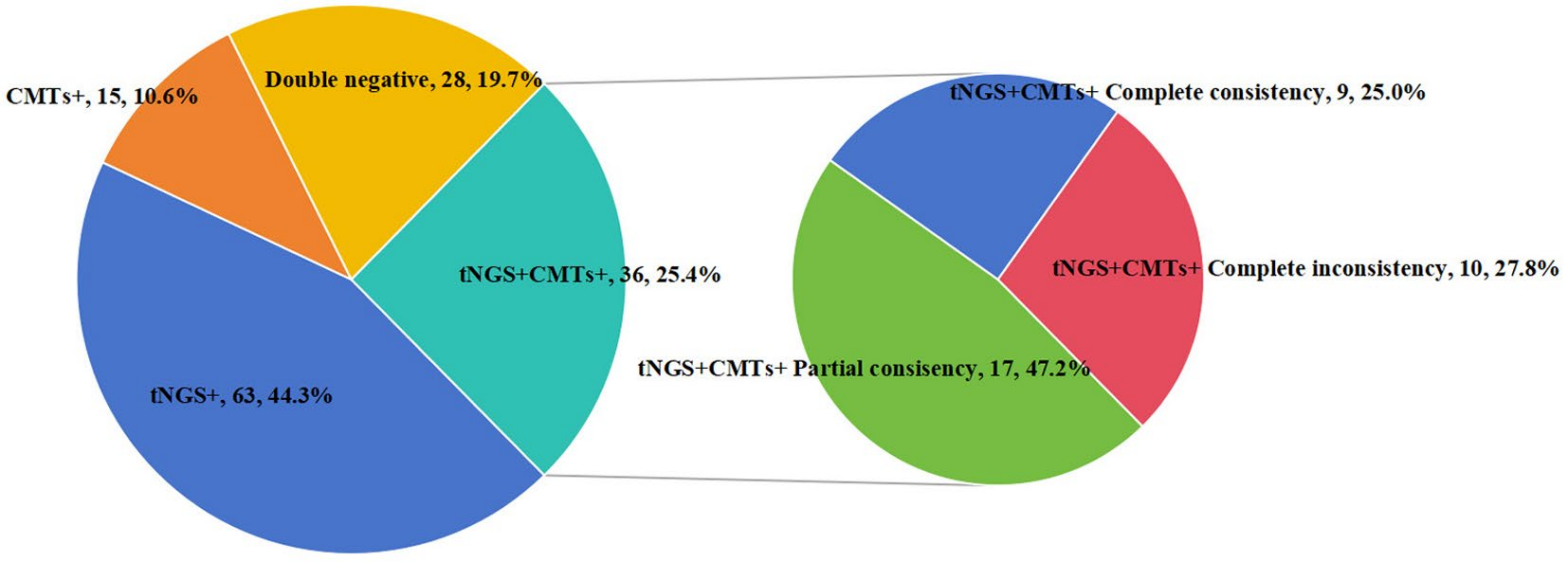
Consistency of pathogen detection results between tNGS and CMTs. Concordance analysis of the results from tNGS and CMTs. Pie chart showing the numbers and proportions of double positive, double negative, and single positive of tNGS and CMTs. Double positive samples were further divided into totally matched, partially matched (at least one same detected pathogen between the two tests), and mismatched groups, and their numbers and proportions were shown in the sub-pie chart.

We analyzed the concordance between tNGS and CMTs. Out of 142 samples, 36 (25.4%) showed positive results for both tNGS and CMTs, while 28 samples (19.7%) were negative in both methods. Furthermore, 63 samples (44.3%) tested positive only with tNGS, and 15 samples (10.6%) were positive exclusively with CMTs. Among the 36 samples positive in both tests, 9 (25.0%) showed complete consistency between tNGS and CMT results, while 17 samples (47.2%) demonstrated partial consistency, and 10 samples (27.8%) showed complete inconsistency (Figure 2).

### 3.4 Distribution of clinically diagnosed pathogens in samples with adult HM patients with infections

**Figure 3:**
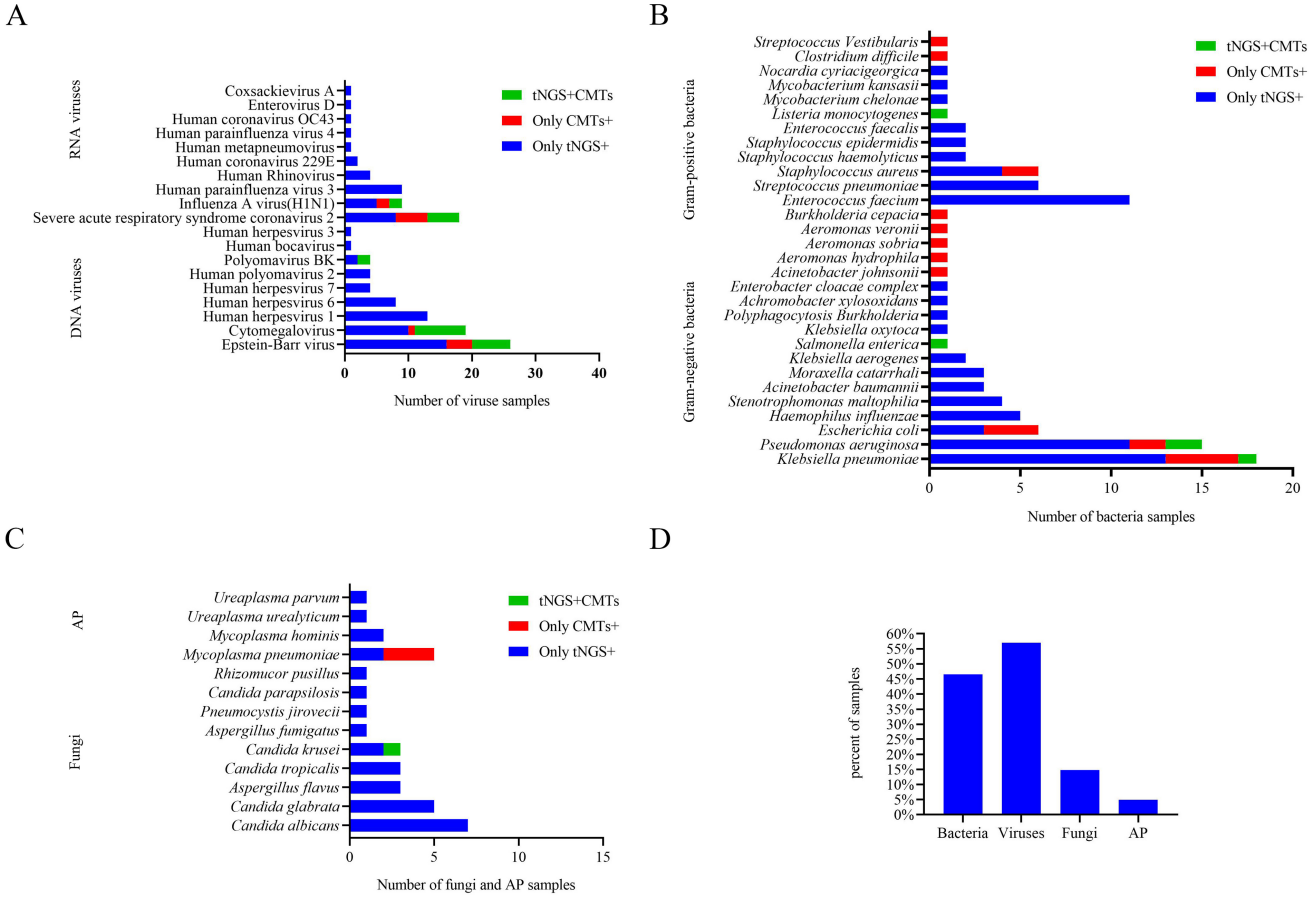
**(A–D)** Distribution of pathogens detected by tNGS and CMTs. AP, atypical pathogens.

Among the 142 samples, a definite or probable microbial etiology of infection was established for 114(80.3%) when tNGS was combined with CMTs. Further analysis of the pathogens detected by tNGS and CMTs showed that the pathogen detection rate for viral infection was the highest (81, 57.0%). Among the viruses detected, the Epstein–Barr virus (DNA-V) was the most frequently detected, accounting for 18.3% of total positive detections, followed by cytomegalovirus (DNA-V, 19, 13.4%), severe acute respiratory syndrome coronavirus 2 (RNA-V, 13, 11.8%), and human herpesvirus 1 (DNA-V, 13, 9.2%). Bacteria were identified in 66 samples (46.5%) by tNGS and CMTs. The detection rate of Gram-negative bacteria (51, 35.9%) was higher than that of Gram-positive bacteria (31, 21.8%) in this study. Among the Gram-negative bacteria, *Klebsiella pneumoniae* (G^−^) had the highest detection rate (18, 12.7%), followed by *Pseudomonas aeruginosa* (G^−^, 15, 10.6%), and *Enterococcus faecium* (G^+^, 11, 7.7%). Other fungi and atypical pathogens were detected in 21 (14.8%) and 7 (4.9%) samples by tNGS and CMTs. The most common species of detected fungi was *Candida*, and the most common species of detected atypical pathogen was *Mycoplasma pneumoniae* (Figure 3).

**Figure 4:**
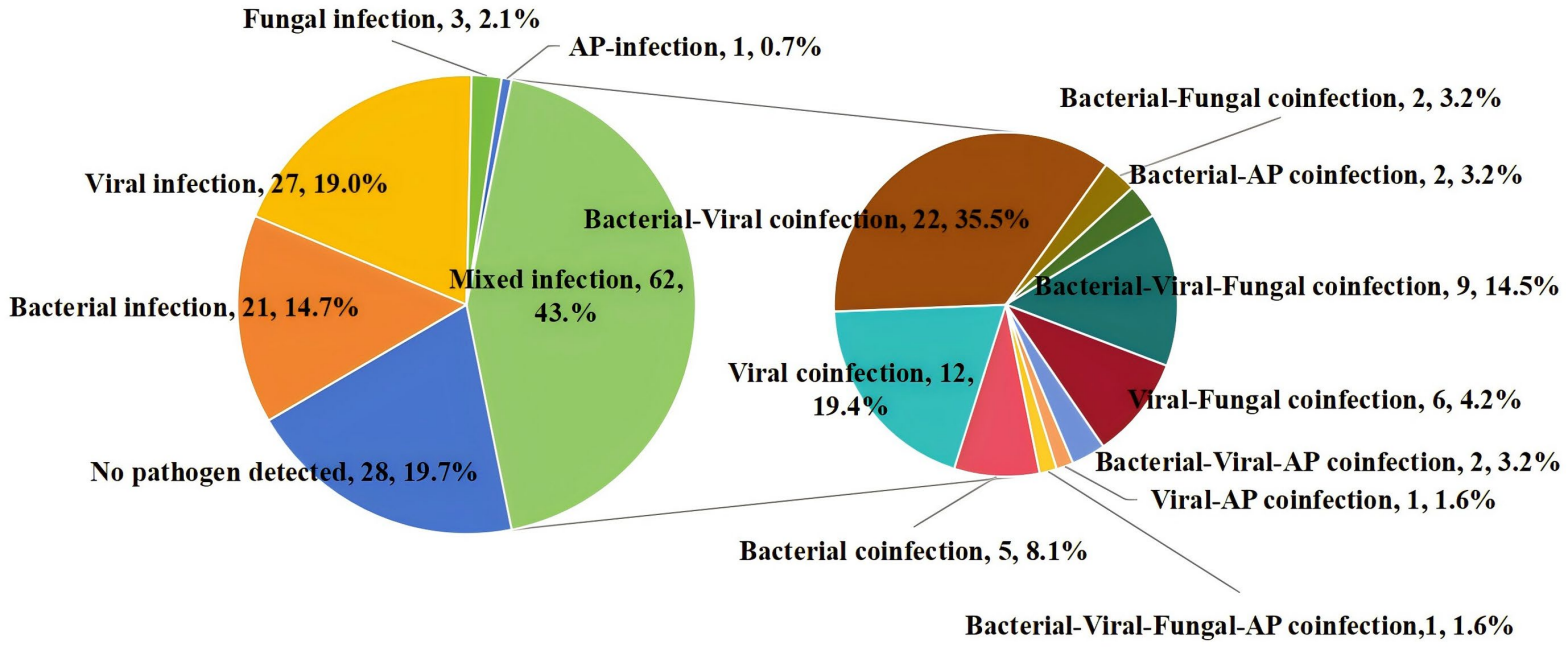
Percentage of patients with mixed infection for various pathogens. AP, atypical pathogens.

Mixed infection was defined as the presence of more than one pathogenic organism in the same sample. By solely utilizing CMTs, only 12 samples (8.5%) were identified as mixed infections. However, when integrating tNGS results, the diagnostic rate of mixed infections increased to 43.7% (62/142). Among the mixed infection types, bacterial-viral co-infection was the most prevalent, accounting for 22 samples (35.5%) (Figure 4).

## 4 Discussion

Infection is a frequent and deadly complication in patients with HM. Effective empiric antibiotic treatment is vital for minimizing the patient’s mortality rate (4). Since identifying the causative pathogen is often not possible, patients may receive prolonged broad-spectrum antibiotic therapy, which fosters the development of resistance. In some cases, the inefficacy or adverse effects of using inappropriate antibiotics could even be lethal. Therefore, there is tremendous room for improvement in the management of patients with HM. One promising strategy is utilizing tNGS for microorganism detection, supplementing standard microbiological tests (3). Recent studies have highlighted the potential of utilizing tNGS for microorganism detection, as it may provide a more comprehensive pathogen profile compared to conventional methods (14, 18, 19). However, challenges such as cost, turnaround time, and detection range limitations remain (16). Our study contributes to this growing body of evidence by comparing tNGS directly with standard microbiological tests in a large cohort of HM patients.

To evaluate the performance of tNGS compared to CMTs, we conducted a single-center study involving 209 hospitalized patients diagnosed with HM, primarily post-high-dose chemotherapy or HSCT. Similar to the findings of Ye et al. (20), our study underscores the utility of tNGS in capturing a broader range of pathogens. Our study is unique compared to others in that it (i) included all types of HM patients, (ii) utilized a retrospective design, (iii) collected all specimens during fever by tNGS, and (iv) compared tNGS with CMTs results obtained within a 48-h window. Additionally, (v) characterized the distribution of pathogens detected by tNGS and CMTs among infected adult HM patients (14, 17, 21, 22). This comprehensive approach aligns with recent trends in precision medicine, where rapid and accurate pathogen identification is critical for tailoring effective treatments.

The cost of a single tNGS test at our institution is 1,462 yuan, while the total cost of a traditional microbiological test ranges from 800 to 1,000 yuan. This indicates that the average cost of tNGS is 1.4 to 1.8 times that of traditional testing. However, the clinical advantages of tNGS, particularly its higher sensitivity and rapid turnaround time, may justify this increased expenditure in certain clinical contexts.

In our study, a total of 61 potential pathogens were detected through tNGS. In comparison, CMTs identified only 21 pathogens. Notably, tNGS, which yielded 60.3% positive results, was found to be more than twice as likely to locate a potential microbiological etiology compared to CMTs, which yielded positive results in only 24.4% of cases. Furthermore, pathogen detection by tNGS resulted in approximately 2-fold higher sensitivity of 69.7% with a high NPV of 48.2% and an overall higher accuracy than CMTs. This enhanced sensitivity and accuracy of tNGS is consistent with other studies in the field. For instance, a study by Ye et al. (20) reported that tNGS identified pathogens in 74.83% of cases where traditional methods, including CMTs, detected pathogens in only 33.11% of cases. Li et al. (23) demonstrated that tNGS has a sensitivity of 81.8%, significantly higher than the 13.6% sensitivity observed with CMTs. These results underscore the superior performance of tNGS in pathogen detection. Although tNGS offers significant advantages in detecting a wide range of pathogens and sensitivity, its limitations warrant careful consideration. Firstly, the detection range of tNGS may not encompass certain specific pathogens, potentially resulting in their undetection. In this study, we identified six microorganisms solely through CMTs—*Clostridium difficile*, *Streptococcus vestibularis*, *Aeromonas hydrophila*, *Aeromonas sobria*, *Aeromonas veronii*, and *Acinetobacter johnsonii*—as these pathogens fell outside the detection capabilities of tNGS. Furthermore, while tNGS was able to detect *Burkholderia cepacia*, it failed to identify this pathogen in certain samples, possibly due to low pathogen concentrations or issues during sample processing. Moreover, our approach to evaluating microbiological results was highly conservative, and we strictly adhered to the paired CMTs. Despite its high sensitivity, tNGS exhibited relatively low specificity in our findings. According to Rossoff et al. (24), NGS achieved a specificity of 59%, compared to 92% (*p* < 0.01) for conventional tests. The specificity of tNGS in our study was 59.7%. Therefore, these results indicate that tNGS is best used in conjunction with CMTs to enhance diagnostic accuracy.

Previous studies have demonstrated that *Klebsiella pneumoniae*, *Enterococcus faecium*, and *Pseudomonas aeruginosa* are commonly predominant bacterial pathogens in patients with HM and infections (2, 4, 24, 25). Similarly, *Pseudomonas aeruginosa*, *Enterococcus faecium*, and *Klebsiella pneumoniae* were the most frequently detected bacterial pathogens using tNGS in our study cohort. Due to their unique immune status, patients with HM may be predisposed to the reactivation of latent viruses, resulting in active infections or even fatal outcomes. Common viral types include Epstein–Barr virus (EBV), cytomegalovirus, and herpes simplex virus (types 1, 6, and 7). The distribution of viral spectrum among positive patients in our study is in line with findings reported by Zhang et al. (4) and Moreno-Sanchez and Gomez-Gomez (2), and Xu et al. (26). In fungal infections, patients with HM subjected to high-intensity chemotherapy exhibit severe immunosuppression, rendering them more susceptible to opportunistic fungal infections. This often results in symptoms that are underrecognized. Previous studies have also noted the challenges in detecting fungal pathogens using traditional methods, which often lack the necessary sensitivity or require specific growth conditions (27, 28). *Candida* species are the most frequently encountered fungi, followed by *Aspergillus* species, with *Aspergillus flavus* being the predominant strain (2). However, *Candida* was the only fungus isolated by the conventional method in our cases. The potential reasons for the low detection rate of fungi may include: (i) traditional fungal culture methods may lack sensitivity to certain fungal species or require specific growth conditions. (ii) Potential inhibitory factors in the samples, such as antibiotics or antifungal medications, could suppress fungal growth, thereby affecting detection outcomes. (iii) The presence of low concentrations of fungi in the samples can complicate detection efforts, as these low levels may be overshadowed by other microorganisms, leading to an increased rate of false negatives (29, 30). Consequently, traditional fungal detection methods may not be sufficiently sensitive or specific. A comprehensive and systematic approach to fungal infection detection, incorporating technologies such as next-generation sequencing (NGS), including tNGS, is required to enhance both sensitivity and specificity. Four atypical pathogens were detected by tNGS in our cases. *Mycoplasma pneumoniae* was the only atypical pathogen isolated by the conventional method. Our results reaffirm that tNGS detection results could furnish pathogen information at the species level, which is crucial for the clinical management of patients with hematological malignancies.

This retrospective analysis of pathogen distribution in samples from adult HM patients experiencing infections aims to gather the main bacterial, viral, atypical pathogen, and fungal agents that affect them and to provide recommendations for formulating initial empiric anti-infective therapy strategies for these patients. Epstein–Barr virus and cytomegalovirus are the viruses of greatest concern. Bacterial infections can be life-threatening and should be considered in any patient presenting with neutropenic fever syndrome. Early initiation of empiric antibiotics, providing coverage against *Klebsiella pneumoniae*, *Pseudomonas aeruginosa*, and Gram-positive cocci, should be started as soon as possible to mitigate complications (31). Fungal infections should be suspected in patients undergoing prolonged chemotherapy who also have persistent fever, and appropriate antifungal coverage against *Candida* spp. and *Aspergillus* spp. should be initiated (32–34). Selection of the initial regimen for atypical pathogens should be based on the patient’s medical history, symptoms, epidemiological data, and CMT results, taking into account the susceptibility patterns of nosocomial pathogens at the institution (2, 35).

Our study is subject to several limitations, including the small sample size and the inherent subjective interpretation of clinically relevant pathogens by treating physicians. Likewise, other peer-reviewed studies encounter similar limitations due to the subjective interpretation of tNGS test data.

In conclusion, the use of tNGS for the rapid identification of pathogens in adult patients with HM and suspected infections offers significant advantages in managing life-threatening infections. Given that the turnaround time for tNGS, including shipping, is 24 to 48 h, its routine use as a complement to standard diagnostics is feasible.

## Data Availability

The original contributions presented in the study are included in the article/Supplementary material, further inquiries can be directed to the corresponding authors.
