# Supplementary material for: Pathogen detection by targeted next-generation sequencing test in adult hematological malignancies patients with suspected infections
Source: Front Med (Lausanne). 2024 Sep 24;11:1443596. doi: 10.3389/fmed.2024.1443596 (PMC11458473; doi:10.3389/fmed.2024.1443596)
Supplement: Supplementary file 1 [file Table_1.DOCX]

# Methods of CMTs

## 1. Microbial culture

Peripheral blood samples were used in aerobic, anaerobic BC bottles of BD BACTEC™ (Becton, Dickinson and Company, Heidelberg, Germany). The minimum incubation time was six days (35 ± 1 °C). Microbial growth was detected in BACTEC FX (Becton, Dickinson and Company, Heidelberg, Germany). BALF, sputum, tissue, and pus samples were cultured on blood agar, chocolate agar, and eosin methylene blue agar at 35°C. All processes were conducted according to the instrument’s standard procedures, and all isolates were identified by Bruker MALDI Biotyper System (Bruker Daltonics Inc, Billerica, MA).

## 2. Real-time PCR

The CMV PCR Kit (20143402167, Daangene, Guangzhou, China), EBV PCR Kit (20173400176, Daangene, Guangzhou, China), BKV PCR Kit (20163402071, Sinomdgene, Beijing, China), JCV PCR Kit (20163402078, Sinomdgene, Beijing, China) and SARS-CoV-2 PCR Kit (20203400749, Daangene, Guangzhou, China) for amplification and quantitation were performed in the ABI 7500 Fast and 7500 Real-Time PCR Systems (Thermo Scientific, Massachusetts, USA) according to the manufacturers’ instructions.

## 3. Serologic testing

The indirect immunofluorescent assay (IFA) PNEUMOSLIDE IgM Kit (Vircell, S.L., Granada, Spain) to simultaneously test antibodies against the main ethiological agents causing infectious: Legionella pneumophila, Mycoplasma pneumoniae, Coxiella burnetii, Chlamydophila pneumoniae, adenovirus, respiratory syncytial virus, influenza A, influenza B and parainfluenza 1, 2 and 3.
