# Supplementary material for: Pathogen detection by targeted next-generation sequencing test in adult hematological malignancies patients with suspected infections
Source: Front Med (Lausanne). 2024 Sep 24;11:1443596. doi: 10.3389/fmed.2024.1443596 (PMC11458473; doi:10.3389/fmed.2024.1443596)
Supplement: Supplementary file 5 [file Table_5.DOCX]

**Supplementary Table S4 Diagnostic Test Evaluation (bacteria and fungus) of 209 samples with tNGS Results**

| Test system | Infection  present/Statistics | Infection not  present/Value |
| --- | --- | --- |
| tNGS positive | 56 | 18 |
| tNGS negative | 19 | 116 |
| MC positive | 24 | 0 |
| MC negative | 51 | 134 |
| tNGS |  |  |
|  | Sensitivity | 74.7% |
|  | Specificity | 86.6% |
|  | Disease prevalence | 35.9% |
|  | Positive Predictive Value | 75.7% |
|  | Negative Predictive Value | 85.9% |
|  | Accuracy (Agreement) | 82.3% |
|  | Kappa | 0.614* |
| Microbial culture |  |  |
|  | Sensitivity | 32.0% |
|  | Specificity | 100.0% |
|  | Disease prevalence | 35.9% |
|  | Positive Predictive Value | 100.0% |
|  | Negative Predictive Value | 72.4% |
|  | Accuracy (Agreement) | 75.6% |
|  | Kappa | 0.376* |

Notes: **p* value < 0.001.

Abbreviations: tNGS, Targeted next generation sequencing; MC, Microbial culture
