# Supplementary material for: Pathogen detection by targeted next-generation sequencing test in adult hematological malignancies patients with suspected infections
Source: Front Med (Lausanne). 2024 Sep 24;11:1443596. doi: 10.3389/fmed.2024.1443596 (PMC11458473; doi:10.3389/fmed.2024.1443596)
Supplement: Supplementary file 6 [file Table_6.DOCX]

**Supplementary Table S5 Diagnostic Test Evaluation (virus) of 209 samples with tNGS Results**

| Test system | Infection  present/Statistics | Infection not  present/Value |
| --- | --- | --- |
| tNGS (virus) positive | 69 | 24 |
| tNGS (virus) negative | 9 | 107 |
| CMTs (virus) positive | 30 | 0 |
| CMTs (virus) negative | 48 | 131 |
| tNGS (virus) |  |  |
|  | Sensitivity | 88.5% |
|  | Specificity | 81.7% |
|  | Disease prevalence | 37.3% |
|  | Positive Predictive Value | 74.2% |
|  | Negative Predictive Value | 92.2% |
|  | Accuracy (Agreement) | 84.2% |
|  | Kappa | 0.675* |
| CMTs (virus) |  |  |
|  | Sensitivity | 38.5% |
|  | Specificity | 100.0% |
|  | Disease prevalence | 37.3% |
|  | Positive Predictive Value | 100.0% |
|  | Negative Predictive Value | 73.2% |
|  | Accuracy (Agreement) | 77.0% |
|  | Kappa | 0.439* |

Notes: **p* value < 0.001.

Abbreviations: tNGS, Targeted next generation sequencing; CMTs, Conventional microbiological tests.

CMTs (virus) include Plasma virus DNA tests and virus NAAT.
